# A Conserved Intramolecular Ion-Pair Plays a Critical but Divergent Role in Regulation of Dimerization and Transport Function among the Monoamine Transporters

**DOI:** 10.3390/ijms25074032

**Published:** 2024-04-04

**Authors:** Sixiang Chen, Xingyu Huang, Xintong Zhang, Chan Li, Yuan-Wei Zhang

**Affiliations:** School of Life Sciences, Guangzhou University, Guangzhou 510006, China; 2112114040@e.gzhu.edu.cn (S.C.); 1112214006@e.gzhu.edu.cn (X.H.); 2112214037@e.gzhu.edu.cn (X.Z.); lichan@gzhu.edu.cn (C.L.)

**Keywords:** monoamine transporters, dimerization, cross-linking, intramolecular ion-pair, transport function, regulation, internalization, transport mechanism

## Abstract

The monoamine transporters, including the serotonin transporter (SERT), dopamine transporter (DAT), and norepinephrine transporter (NET), are the therapeutic targets for the treatment of many neuropsychiatric disorders. Despite significant progress in characterizing the structures and transport mechanisms of these transporters, the regulation of their transport functions through dimerization or oligomerization remains to be understood. In the present study, we identified a conserved intramolecular ion-pair at the third extracellular loop (EL3) connecting TM5 and TM6 that plays a critical but divergent role in the modulation of dimerization and transport functions among the monoamine transporters. The disruption of the ion-pair interactions by mutations induced a significant spontaneous cross-linking of a cysteine mutant of SERT and an increase in cell surface expression but with an impaired specific transport activity. On the other hand, similar mutations of the corresponding ion-pair residues in both DAT and NET resulted in an opposite effect on their oxidation-induced dimerization, cell surface expression, and transport function. Reversible biotinylation experiments indicated that the ion-pair mutations slowed down the internalization of SERT but stimulated the internalization of DAT. In addition, cysteine accessibility measurements for monitoring SERT conformational changes indicated that substitution of the ion-pair residues resulted in profound effects on the rate constants for cysteine modification in both the extracellular and cytoplasmatic substrate permeation pathways. Furthermore, molecular dynamics simulations showed that the ion-pair mutations increased the interfacial interactions in a SERT dimer but decreased it in a DAT dimer. Taken together, we propose that the transport function is modulated by the equilibrium between monomers and dimers on the cell surface, which is regulated by a potential compensatory mechanism but with different molecular solutions among the monoamine transporters. The present study provided new insights into the structural elements regulating the transport function of the monoamine transporters through their dimerization.

## 1. Introduction

The neurotransmitter sodium symporters (NSSs) comprise a group of transporters responsible for the reuptake of neurotransmitters, such as monoamines and amino acids. Among these NSS members, monoamine transporters, including the serotonin transporter (SERT), dopamine transporter (DAT), and norepinephrine transporter (NET), constitute a subgroup in the NSS family, which terminate monoaminergic signaling by the reuptake of monoamine neurotransmitters from the synaptic cleft after their secretion and play critical roles in monoamine neurotransmission in the central nervous system (CNS). Notably, these monoamine transporters are the therapeutic targets for the treatment of many neuropsychiatric disorders, such as depression, anxiety, and attention deficit hyperactivity disorder [1,2,3,4], and also the molecular targets for drugs of abuse, such as cocaine and amphetamines [5,6].

The monoamine transporters share a common structural fold comprising 12 transmembrane domains (TM1-12) and a similar transport mechanism by which they utilize transmembrane ion gradients of Na^+^ and Cl^−^ to drive their respective substrates across the membrane [7,8]. The recently resolved high-resolution structures of DAT and SERT in several conformational states have revealed the common binding sites for their substrates and ions and provided insights into the conformational changes that occur during the transport process [9,10,11,12,13]. Despite significant progress in characterizing the structures and transport mechanisms of the monoamine transporters, many aspects of the mechanism of regulation of their transport activities remain to be understood. One challenge is to reveal the mechanism of transporter dimerization or oligomerization and its significance in the regulation of transport function [14,15].

Earlier biochemical studies have demonstrated the presence of dimers and higher-order oligomers of the monoamine transporters in the plasma membrane [16,17,18]. Although the leucine transporter (LeuT), an amino acid transporter in the NSS family, has been crystallized as a dimer with TM9 and TM12 in the dimeric interface [19], a similar dimeric architecture has not been observed in the monoamine transporters. The high-resolution structures of SERT and DAT showed a pronounced kink in the middle of TM12 [9,12], suggesting that they form dimers by a mechanism different from that of LeuT [15]. By comparison, in DAT, an endogenous cysteine residue Cys306, located at the end of the third extracellular loop (EL3) connecting TM5 and TM6, has been demonstrated to be involved in the interfacial interactions in its dimerization and to be responsible for intermolecular disulfide bond formation upon oxidative treatment [20], indicating the presence of different dimeric quaternary structures in the NSS transporters.

Oxidation-induced dimerization of DAT has been shown to inactivate its transport activity [21], suggesting that interfering with the interfacial interactions between protomers can result in a profound effect on its transport function. Previously, a highly conserved intramolecular ion-pair in DAT, R304-E307, was proposed to be critical for stabilizing EL3 in a conformation that modulates its dimerization and transport function [22]. Experimental results showed that mutations of the ion-pair residues in DAT led to a dramatic reduction in cell surface expression but with an increased specific transport activity [23]. The mechanism underlying the reciprocal correlation between the dimerization and transport function of DAT has been speculated to be conformational restriction occurring in its dimers in the transport cycle [24]. These studies, however, did not address the mechanistic details by which the conserved ion-pair, as an important structural factor, regulates the transport function through its dimerization.

Recently, we characterized the conserved ion-pair in the regulation of dimerization and transport function of GlyT1, an amino acid transporter in the NSS family [25,26]. Our results showed that similar substitutions of the corresponding ion-pair residues in GlyT1 resulted in an increase in both dimerization and cell surface expression but with an impaired transport activity. The opposite effects of the ion-pair on DAT and GlyT1 raise the question of what reasons cause the different influences of the ion-pair interactions on their transport function and whether all monoamine transporters share a unifying mechanistic feature for the ion-pair-mediated regulation of dimerization and transport functions.

In the present study, we expand our study to all monoamine transporters to investigate the influence of the conserved intramolecular ion-pair on dimerization, endocytosis, and the transport activity of SERT, DAT, and NET. Our results show that the ion-pair plays a critical but divergent role in the regulation of dimerization and transport function among these transporters. The present study provides new insights into the structural elements regulating the transport function of the monoamine transporters through their dimerization.

## 2. Results

### 2.1. Dimer Models of Monoamine Transporters

**Figure 1:**
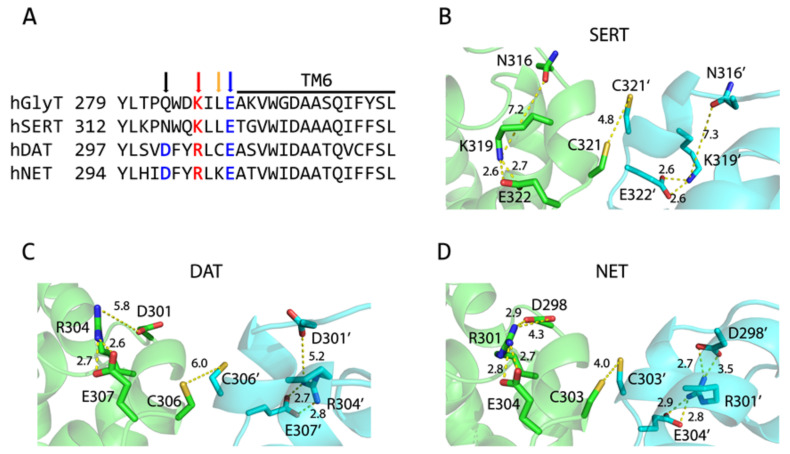
Amino acid sequence alignment and dimer models of the monoamine transporters. (**A**) Amino acid sequence alignment of the EL3 of human monoamine transporters (SERT, DAT, and NET) and the glycine transporter GlyT1. Each line represents a different sequence with the residue number preceding the sequence. The positions where an endogenous Cys306 in DAT and the corresponding residues mutated to cysteine in SERT and NET are marked with a yellow arrow. The conserved ion-pair residues are in red (positively charged) or blue (negatively charged) letters and marked with an arrow in the same color, respectively. An additional aspartate residue that is present only in DAT or NET is in blue letters and marked with a black arrow. (**B**–**D**) Dimer models of the monoamine transporters, SERT (**B**), DAT (**C**), and NET (**D**) with each protomer in an outward open conformation. An intramolecular salt bridge formed by K319-E322 (SERT), R304-E307 (DAT), or R301-E304 (NET) in the EL3 near the extracellular end of TM6 is shown in each protomer, respectively. An additional ionic interaction, formed by D301-R304 in DAT or D298-R301 in NET, is also shown. The two protomers in a putative SERT L321C, DAT, or NET K303C dimer are colored green and cyan, respectively.

According to cross-linking experiments with DAT, the endogenous cysteine residue Cys306 was demonstrated to be responsible for oxidation-induced dimerization, which led to a profound loss of its transport activity (21, Figure 1A). Similar results were also observed with oxidative cross-linking of the corresponding GlyT1 cysteine mutant L288C [26], suggesting that the residues at the equivalent position (marked by a yellow arrow in Figure 1A) in other monoamine transporters, SERT and NET, could also participate in the interfacial interactions in their respective dimers. Thus, it is expected that an oxidative cross-linking strategy can be used for monitoring the interfacial interactions and the influences of dimerization on the transport function of the monoamine transporters.

In our dimer models of the monoamine transporters (DAT WT, SERT L321C, and NET K303C), the corresponding cysteines between two protomers are in close proximity, only 4–6 Å apart, allowing the possibility of disulfide bond formation upon oxidation (Figure 1B–D). It is notable that there is a highly conserved intramolecular ion-pair (marked by red and blue arrows in Figure 1A, K319-E322 in SERT, R304-E307 in DAT, or R301-E304 in NET) which can form a salt bridge that is expected to stabilize the EL3 in a conformation that keeps the two cysteine residues at a cross-linking distance (Figure 1A–D). Strikingly, an additional ionic interaction formed by the carboxyl group of an N-terminal adjacent aspartate residue (marked by a black arrow in Figure 1A) with the guanidino group of an arginine residue (D301-R304 in DAT or D298-R301 in NET) is also present in DAT and NET, but not in SERT, in which the corresponding residue is Asn316 and is more than 7 Å away from K319 (Figure 1B–D).

### 2.2. Oxidative Cross-Linking of SERT L321C and Its Functional Consequences

**Figure 2:**
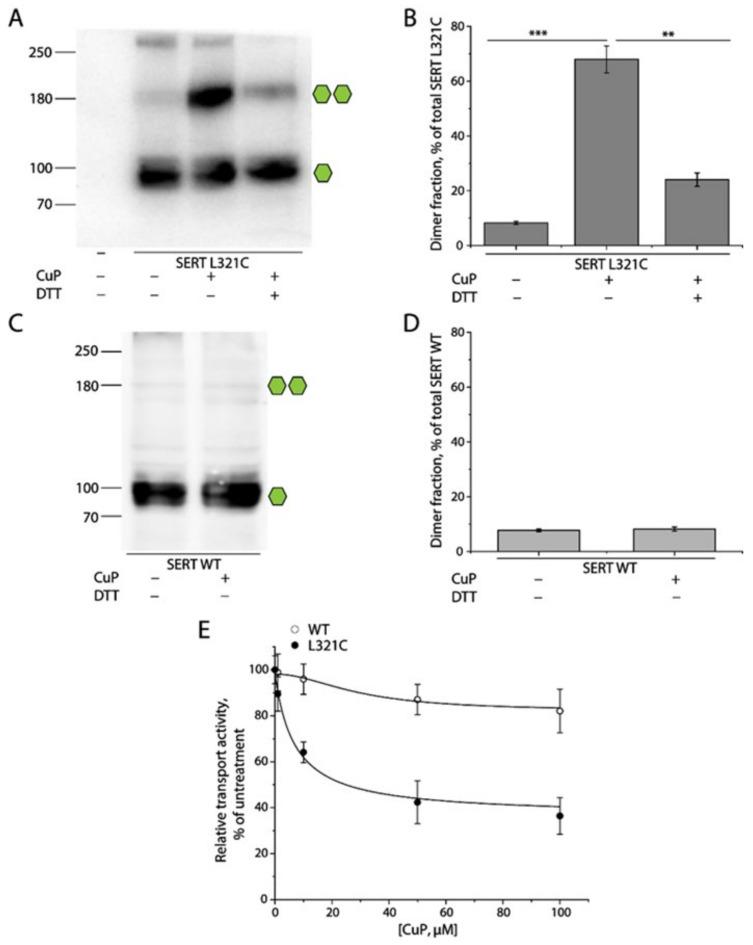
Oxidative cross-linking of SERT L321C and its functional consequences. (**A**,**C**) Representative immunoblots of SERT L288C (**A**) or WT (**C**) with or without CuP treatment. Numbers on the left of each blot represent molecular masses in kDa of protein standards. Single or double green hexagons represent the fully glycosylated monomers or dimers, respectively. (**B**,**D**) Quantification of dimer fraction in total SERT. The fully glycosylated dimers (−180 kDa) were considered to reside on the cell surface, whereas the non-glycosylated dimers were within the cytoplasm. Dimer fraction was expressed as an integrated density percentage of the fully glycosylated dimers in total SERT. Results are shown as mean ± SEM (n = 3). ** *p* < 0.01; *** *p* < 0.001. (**E**) The effects of CuP concentrations on the transport activity of SERT WT or L321C mutant. APP^+^ uptake by SERT WT or L321C mutant was expressed as a percentage of the value obtained without CuP treatment. All results are presented as mean ± SEM (n = 3).

To examine the intermolecular interactions between SERT protomers, we substituted Leu321 with cysteine and monitored the oxidative cross-linking of the L321C mutant and its influence on both dimerization and transport function. HEK 293T cells stably expressing SERT L321C or WT were treated with an oxidative cross-linker, copper phenanthroline (CuP), and lysed with RIPA buffer. The lysates were then subjected to an immunoblot analysis for CuP-induced SERT dimerization (Figure 2). The L321C mutant without CuP treatment was present as fully glycosylated monomers (~90 kDa) with a trace of fully glycosylated dimers (~180 kDa) and a small portion of oligomers (more than 250 kDa) when measured in total HEK293 cell lysates (Figure 2A, lane 2). CuP treatment significantly increased the amounts of fully glycosylated dimers, at ~180 kDa (Figure 2A, lane 3), and the dimer fraction, in total L321C lysates (Figure 2B, middle column). The cross-linked dimer fraction of L321C at 100 μM CuP was estimated to be approximately 59.63 ± 5.44% of the total number of SERTs in lysates. In addition, the CuP-induced dimerization of the L321C mutant was dramatically decreased by DTT treatment (Figure 2A, lane 4 and Figure 2B, right column), indicating that CuP oxidation induced the intermolecular Cys321-Cys321′ disulfate bond formation between L321C protomers (Figure 1B). As control experiments, we examined the effect of CuP treatment on WT dimerization. CuP treatment had little effect on the distribution pattern and dimer fraction of SERT WT in our immunoblot analysis (Figure 2C,D).

We then evaluated the influence of CuP oxidation on the transport activity of SERT WT and the L321C mutant. Transport activity after CuP treatment at various concentrations was measured by using a fluorescent substrate, APP^+^. Our results indicated that CuP treatment progressively decreased substrate transport by SERT L321C in a CuP concentration-dependent manner (Figure 2E). The transport activity remaining at the highest CuP concentration (100 μM) was estimated to be 32.10 ± 4.38% of the control measurements, in the absence of CuP, consistent with the uncross-linked fraction (monomers and natural dimers) in the total lysates (Figure 2B, lanes 1 and 2). The CuP concentration leading to a half-maximal inactivation was 14.63 ± 2.82 μM. The CuP cross-linked SERT dimers, thus, were considered to be inactive in transporting substrate. By contrast, CuP treatment had little effect on substrate transport by SERT WT (Figure 2E). These results on SERT dimerization and its functional response suggest that it is appropriate to assess the intermolecular interactions between protomers by examining CuP-induced cross-linking and its influence on the transport activity of a SERT mutant with a cysteine residue placed in the SERT dimeric interface.

### 2.3. Influence of the Intramolecular K319-E322 Ion-Pair on CuP Cross-Linking of SERT L321C

**Figure 3:**
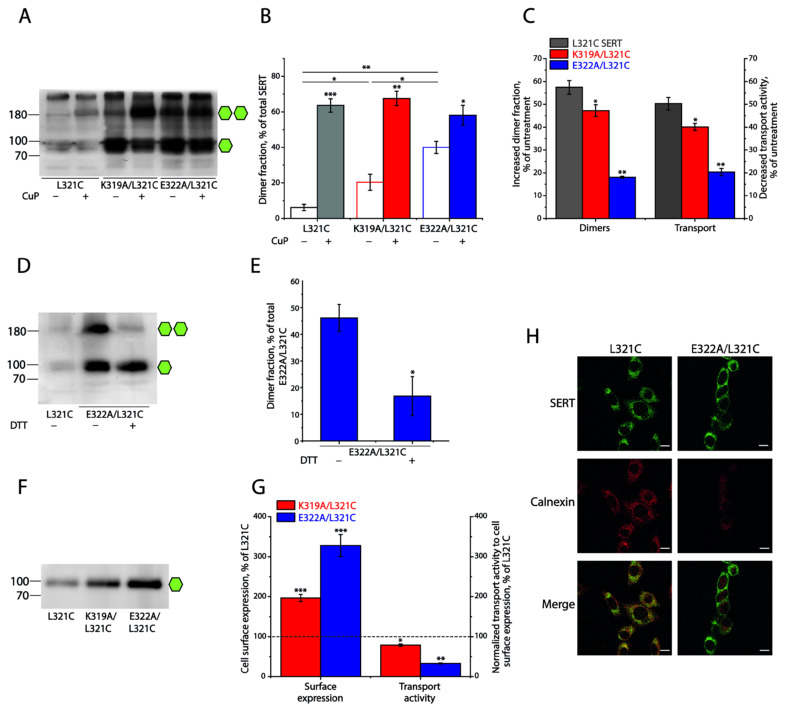
The intramolecular ion-pair (K319-E322) in the EL3 modulates dimeric interface interactions and transport activity of SERT L321C. Numbers on the left of each blot represent molecular masses in kDa of protein standards. Single or double green hexagons represent the fully glycosylated monomers or dimers, respectively. (**A**) A representative immunoblot of the ion-pair mutants K319A/L321C or E322A/L321C with or without CuP treatment. (**B**) Quantification of dimer fractions of SERT L321C, K319A/L321C, and E322A/L321C with or without CuP treatment. The dimer fraction was expressed as a percentage of the fully glycosylated dimers in total SERT. Results are presented as mean ± SEM (n = 3). * *p* < 0.05; ** *p* < 0.01; *** *p* < 0.001 compared to each mutant without CuP treatment or between the indicated samples. (**C**) Transport activities of SERT mutants. Relative APP^+^ uptake of each mutant was expressed as a percentage of transport activity without CuP treatment. All results are presented as mean ± SEM (n ≥ 3). * *p* < 0.05; ** *p* < 0.01 compared to each mutant without CuP treatment. (**D**) A representative immunoblot of the SERT E322A/L321C mutant with or without DTT treatment. (**E**) Quantification of the dimer fraction of SERT E322A/L321C with or without DTT treatment. Dimer fraction was expressed as a percentage of the fully glycosylated dimers relative to the total SERT E322A/L321C. Results are presented as mean ± SEM (n = 3). * *p* < 0.05 compared to SERT E322A/L321C without DTT treatment. (**F**) A representative immunoblot of biotinylated SERT L321C, K319A/L321C, and E322A/L321C. (**G**) Quantification of cell surface expression and transport activity of SERT L321C, K319A/L321C, and E322A/L321C. Cell surface expression was expressed as a percentage of the integrated density of SERT L321C. Transport activity was expressed as a percentage of APP^+^ uptake normalized to cell surface expression of SERT L321C. The dotted line shows cell surface expression and transport activity of the SERT background construct, L321C. * *p* < 0.05; ** *p* < 0.01; *** *p* < 0.001 compared to the background construct, L321C. (**H**) Colocalization of SERT immunoreactivity with ER marker calnexin. Confocal images of HeLa cells stably expressing SERT constructs (L321C, left panels; E322A/L321C, right panels) immunostained with antibodies to SERT (green) and calnexin (red) (scale bar: 10 μm). The double immunofluorescence staining experiments were performed three times with similar results.

To examine the significance of the conserved intramolecular ion-pair in the regulation of the interfacial interactions between SERT protomers, we substituted each of the ion-pair residues with alanine, one at a time, in the L321C background, and investigated the effects of these substitutions on CuP oxidative cross-linking and transport function. As shown in Figure 3A,B, compared to the background L321C, disruption of the ion-pair interactions by mutations led to a remarkably spontaneous cross-linking, even without CuP treatment in either K319A/L321C or E322A/L321C mutant, of which E322A/L321C apparently had a larger fraction of spontaneously cross-linked dimers than K319A/L321C. While CuP treatment further increased cross-linking of K319A/L321C or E322A/L321C to a total cross-linking level (sum of spontaneous cross-linking and CuP-induced cross-linking) similar to that of L321C (Figure 3B), the CuP-induced dimer fraction of K319A/L321C or E322A/L321C was smaller than that of its background construct (Figure 3C). Consequently, the increased dimer fraction resulting from CuP cross-linking in these mutants led to a proportional reduction in their transport activities (Figure 3C). It is notable that a small portion of oligomers (more than 250 kDa) with similar levels are present in all SERT constructs, including the background L321C, but CuP treatment had little effect on their immunoreactivities (Figure 3A). Although we do not exactly know how they form, the naturally occurring oligomerization is unlikely to be influential to our investigation of CuP-induced dimerization and its functional consequences. Figure 3D,E shows that DTT treatment remarkably reduced the dimer fraction in the immunoblot analysis for E322A/L321C, indicating a spontaneous intermolecular Cys321-Cys321′ disulfide bond formation in this ion-pair mutant. These results suggest that the prevention of the ion-pair residues to form a salt bridge by mutations in SERT leads to an increase in the interfacial interactions, which, in turn, induces a spontaneous cross-linking of SERTs into dimers.

It is noticeable that the ion-pair mutants, either K319A/L321C or E322A/L321C, had a stronger SERT band density in the immunoblot analysis for total lysates, compared to its background, L321C (Figure 3A). We then performed biotinylation experiments to determine their expression levels on the cell surface. As shown in Figure 3F,G, the cell surface expression level of K319A/L321C or E322A/L321C was 1.9- or 3.2-fold higher than that of its background, L321C, respectively, suggesting that these mutations led to a dramatic increase in the cell surface expression of SERT. The two ion-pair mutants, K319A/L321C and E322A/L321C, however, did not show a proportional increase in transport activity, as indicated by an increase in their cell surface expression. On the contrary, the transport activity of K319A/L321C or E322A/L321C normalized to its cell surface expression (specific transport activity) was decreased to only 78% or 32% of the L321C background, respectively (Figure 3G). In addition, our steady-state kinetic analysis indicated that these ion-pair mutants showed comparable *K_m_* and *V_max_* values for substrate transport to those of SERT WT and that CuP-induced cross-linking significantly reduced their *V_max_* but had little effect on *K_m_* for the substrate APP^+^ (Supplementary Table S1), suggesting that dimerization of SERT does not alter its substrate binding affinity.

To validate the ion-pair mutation-induced increase in the cell surface expression of SERT, we further examined the cellular distribution of E322A/L321C and its background, L321C, by using a double immunofluorescence staining approach. As shown in Figure 3H, the background L321C immunoreactivity was observed throughout the cell and surface in confocal images, showing extensive overlap with the reactivity of the intracellular endoplasmic reticulum (ER) marker calnexin (left panels). In contrast, E322A/L321C immunoreactivity was seen predominantly on the cell membrane, while little overlap between the E322A/L321C and ER markers occurred (right panels), consistent with our biotinylation results. These results suggest that elimination of the K319-E322 ionic interactions promotes interfacial interactions between SERT protomers, resulting in an increased expression of the transporter on the cell surface, but consequently causing impairment of its transport function.

### 2.4. Effects of the Ion-Pair Interactions on DAT

**Figure 4:**
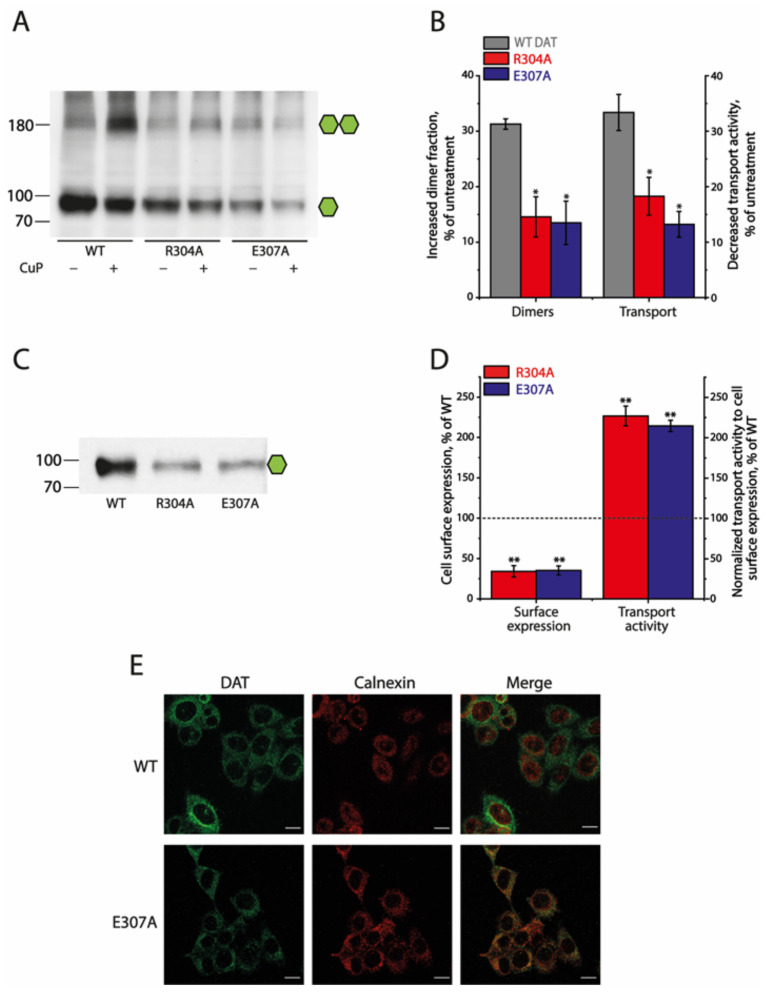
Effects of the ion-pair mutations on the CuP-induced dimerization, transport activity, and cell surface expression of DAT. The numbers on the left of each blot represent molecular masses in kDa of protein standards. Single or double green hexagons represent the fully glycosylated monomers or dimers, respectively. (**A**) A representative immunoblot of DAT WT and its ion-pair mutants with or without CuP treatment. (**B**) Quantification of CuP-induced increases in dimer fraction and decreases in transport activity of DAT WT and its mutants. Increased dimer fraction or decreased transport activity by CuP treatment was calculated from the control dimer fraction or transport activity without CuP treatment by subtracting its respective value after CuP treatment, respectively. Results are presented as mean ± SEM (n = 3). * *p* < 0.05 compared to DAT WT. (**C**) A representative immunoblot of the cell surface expression of DAT WT and its mutants. (**D**) Quantification of cell surface expression and normalized transport activity of WT DAT and its mutants. Cell surface expression of DAT mutants was expressed as a percentage of the integrated density of DAT WT detected by the biotinylation experiments. Transport activity was expressed as a percentage of ASP^+^ uptake normalized to the cell surface expression of DAT WT. The dotted line shows the cell surface expression and transport activity of DAT WT. Results are presented as mean ± SEM (n = 3). ** *p* < 0.01, compared to DAT WT. (**E**) Colocalization of DAT immunoreactivity with the ER marker calnexin. Confocal images of HeLa cells stably expressing DAT constructs (WT, upper panels; E307A, lower panels) immunostained with antibodies to DAT (green) and calnexin (red) (scale bar: 10 μm). The double immunofluorescence staining experiments were performed three times with similar results.

To compare the effects of the conserved ion-pair interactions on SERT and DAT, we substituted the corresponding residues, similarly to what we did with SERT, and monitored the effects of these mutations on both the dimerization and transport function of DAT, as described previously [22]. CuP treatment increased the dimer fraction of DAT WT or its mutants (Figure 4A,B). Unlike the SERT mutants, little spontaneous cross-linking caused by the ion-pair mutations was seen in DAT without CuP treatment. In addition, it is notable that CuP treatment led to both an increase in oxidative cross-linking and a reduction in the transport activity of the ion-pair mutants to extents that were significantly less than those of WT (Figure 4A,B), suggesting that disruption of the ion-pair interactions results in an impairment of the interfacial interactions between DAT protomers and a reduction in the CuP inactivation of transport function. Furthermore, oxidative cross-linking decreased *V_max_* but with little effect on *K_m_* values for the substrate ASP^+^ of the DAT ion-pair mutants (Table S2), consistent with the effects of CuP on the kinetic parameters of the corresponding mutants of SERT.

Substitutions of the ion-pair residues dramatically reduced DAT cell surface expression. Biotinylation experiments showed that the cell surface expression levels of R304A and E307A were only 34.12 ± 7.07% or 35.14 ± 5.56% of that of DAT WT, respectively (Figure 4C,D). In addition, our immunofluorescence analysis for the colocalization of DAT immunoreactivity with the ER marker calnexin indicated that WT immunoreactivity was seen predominantly on the cell membrane with little colocalization with calnexin (Figure 4E upper panels), but E307A immunoreactivity was seen internally, with a pattern very similar to that seen for the intracellular calnexin (Figure 4E, lower panels), as opposed to that between WT DAT immunoreactivity and calnexin. Strikingly, the specific transport activities of these ion-pair mutants normalized to their cell surface expressions were increased by more than 2-fold compared to that of DAT WT (Figure 4D). These results suggest that elimination of the ion-pair interactions by mutations reduces the interfacial interactions between DAT protomers, resulting in a decreased expression on the cell surface with an increased specific transport activity, indicating an opposite effect of the conserved ion-pair on DAT compared to that on SERT.

### 2.5. Effects of the Ion-Pair Interactions on NET

**Figure 5:**
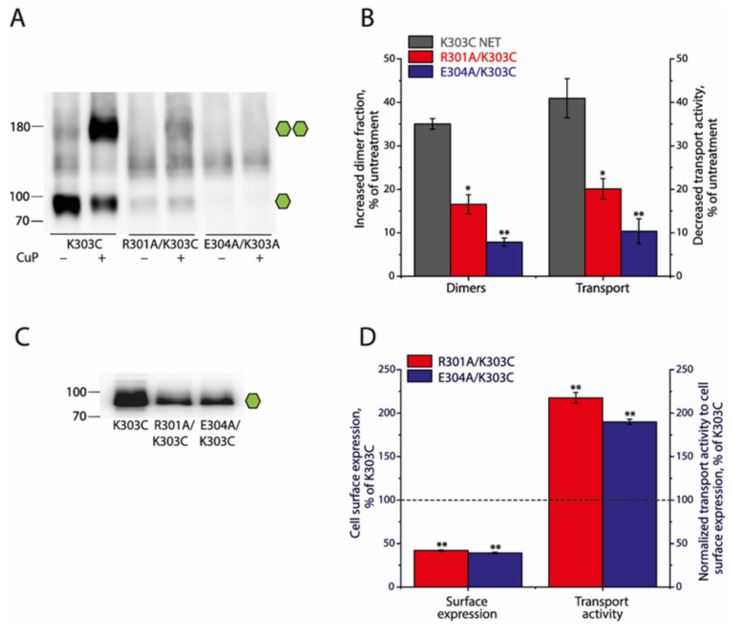
Effects of the ion-pair mutations on the CuP-induced dimerization, transport activity, and cell surface expression of the NET. Numbers on the left of each blot represent molecular masses in kDa of the protein standards. Single or double green hexagons represent the fully glycosylated monomers or dimers, respectively. (**A**) A representative immunoblot of the background, K303C of NET, and its ion-pair mutants with or without CuP treatment. (**B**) Quantification of the CuP-induced increases in dimer fraction and decreases in transport activity of NET background K303C and its mutants. The increased dimer fraction or decreased transport activity by CuP treatment was calculated from the control dimer fraction or transport activity without CuP treatment by subtracting its respective value after CuP treatment, respectively. Results are presented as mean ± SEM (n = 3). * *p* < 0.05; ** *p* < 0.01 compared to the NET background K303C. (**C**) A representative immunoblot of the cell surface expression of NET background K303C and its mutants. (**D**) Quantification of cell surface expression and normalized transport activity of NET background K303C and its mutants. The cell surface expression of NET ion-pair mutants was expressed as a percentage of the integrated density of the NET background K303C. Transport activity was expressed as a percentage of ASP^+^ uptake normalized to the cell surface expression of NET background K303C. The dotted line shows the cell surface expression and transport activity of NET background K303C. All results are presented as mean ± SEM (n = 3). ** *p* < 0.01, compared to NET background K303C.

It is expected that the NET shares a similar mechanism of dimerization-mediated regulation of its transport function to DAT, due to a high degree of amino acid sequence identity between both (~80%). To confirm the effects of the ion-pair interactions on the NET, we substituted the ion-pair residues with alanine in a background construct, K303C, in which cysteine was placed at the equivalent position of Cys306 in DAT (Figure 1A). As shown in Figure 5A,B, CuP treatment increased NET dimerization but disruption of the ion-pair interactions impaired its CuP-induced dimerization and inactivation of substrate transport. Similar to the results with SERT and DAT, NET dimerization had little effect on the substrate binding affinity (Table S3). In addition, while the cell surface expression levels of R301A/K303C and E304A/K303C were 41.95 ± 0.88% and 39.13 ± 0.86% of the background, K303C, the specific transport activity, normalized to its cell surface expression, was estimated to be 217.53 ± 6.30% or 190.09 ± 2.95% of K303C, respectively (Figure 5C,D), validating a similar effect of the ion-pair interactions on NET to its effect on DAT.

### 2.6. Effects of the Ion-Pair Interactions on Internalization (Endocytosis) of SERT and DAT

To understand the divergent effects of the ion-pair on SERT and DAT, we performed reversible biotinylation experiments to investigate the internalization of the ion-pair mutants. The internalization of biotinylated cell surface-resident SERTs or DATs was performed at 37 °C for 30 min and terminated by adding an ice-cold buffer. After biotins from the cell surface-resident proteins were cleaved by incubating with a membrane-impermeant reducing agent, MesNa, the internalized biotinylated SERTs or DATs, defined as the amounts of MesNa-resistant SERTs or DATs, were captured by streptavidin beads from total lysates and quantified by immunoblot analysis.

**Figure 6:**
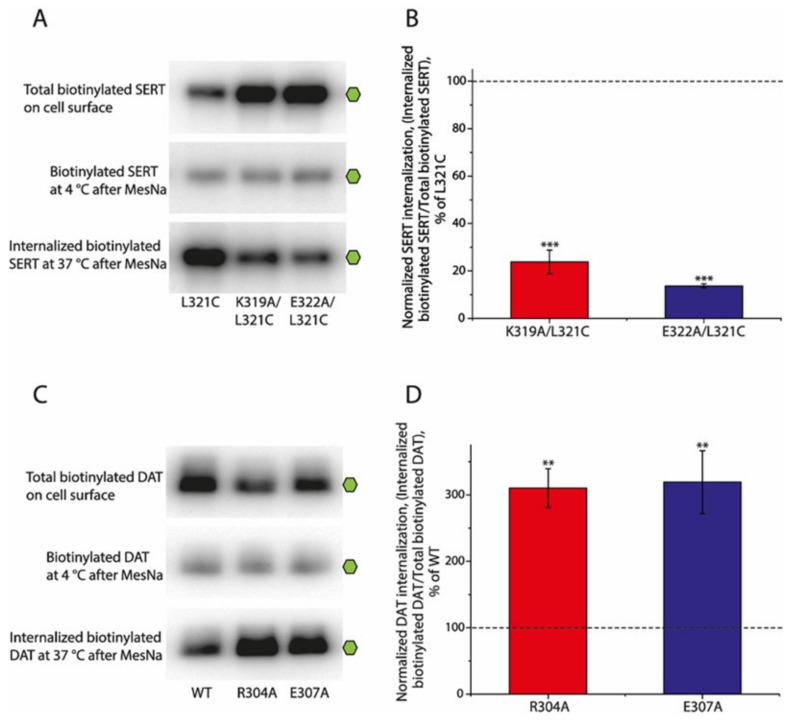
Effects of the ion-pair mutations on the internalization of SERTs and DATs. The single green hexagon on the right of each blot represents the fully glycosylated monomers. (**A**) Representative immunoblots of total biotinylated SERTs on the cell surface, biotinylated SERTs on the cell surface at 4 °C after MesNa treatment, and internalized biotinylated SERTs at 37 °C after MesNa treatment. (**B**) Normalized SERT internalization. Internalized biotinylated SERTs captured by streptavidin beads after subtracting MesNa-resistant biotinylated SERTs were normalized to the total biotinylated SERTs on the cell surface. Results are presented as mean ± SEM (n = 3). *** *p* < 0.001 compared to SERT background control shown by the dotted line. (**C**) Representative immunoblots of the total biotinylated DAT on the cell surface, biotinylated DAT on the cell surface at 4 °C after MesNa treatment, and internalized biotinylated DAT at 37 °C after MesNa treatment. (**D**) Normalized DAT internalization. Internalized biotinylated DAT captured by streptavidin beads after subtracting MesNa-resistant biotinylated DAT was normalized to its total biotinylated DAT on the cell surface. Results are presented as mean ± SEM (n = 3). ** *p* < 0.01 compared to DAT WT control shown by the dotted line.

The ion-pair mutants of SERT, K319A/L321C and E322A/L321C, showed a significant increase in their cell surface expression, revealed by total biotinylated SERTs on the cell surface without MesNa treatment, compared to that of their background construct, L321C (Figure 6A, upper panel). By comparison, similar substitutions in DAT, R304A and E307A, led to a remarkable decrease in their cell surface expression (Figure 6C, upper panel). MesNa treatment immediately after biotinylation at 4 °C dramatically reduced the amounts of biotinylated SERTs or DATs to a similar level as that of the uncleaved SERT or DAT mutants to that of their respective background controls (Figure 6A,C, middle panels), confirming that MesNa can efficiently reverse the biotinylation of SERTs or DATs. Interestingly, similar substitutions of the ion-pair residues led to an opposite effect on the internalization of SERTs or DATs. As shown in Figure 6A, the amounts of internalized biotinylated SERTs captured by the streptavidin beads in the total lysates of the ion-pair mutants after MesNa treatment were significantly lower than those in its background (lower panel). Because of their higher cell surface expression levels than those of WT, the normalized internalizations of the ion-pair mutants, K319A/L321C and E322A/L321C, expressed as a percentage of internalized biotinylated SERTs over total biotinylated SERTs on the cell surface, were estimated to be only 23.82 ± 5.01% and 13.68 ± 0.86% of WT, respectively (Figure 6B), indicating that substitutions of the ion-pair residues lead to an increased cell surface expression with a decreased internalization of SERTs. By contrast, although the ion-pair mutants of DAT showed a lower cell surface expression than that of the WT, the amounts of internalized biotinylated DAT in these mutants were remarkably increased compared to those of the WT (Figure 6C, lower panel). Correspondingly, the normalized internalizations of the ion-pair mutants of DAT, R303A and E307A, were estimated to be 310.05 ± 29.28% and 319.14 ± 47.43% of those of the WT, respectively (Figure 6D), indicating that similar substitutions of the ion-pair residues result in a decreased cell surface expression with an increased internalization of DAT.

### 2.7. Effects of Substitutions of Asp301 on DAT

**Figure 7:**
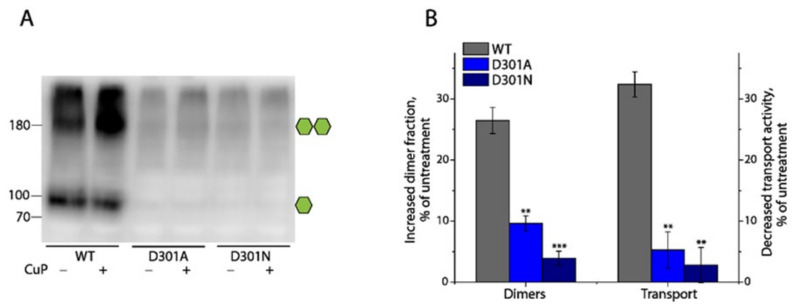
Effects of substitutions of Asp301 on CuP-induced dimerization and transport activity of DAT. Numbers on the left of the immunoblot represent molecular masses in kDa of protein standards. Single or double green hexagons represent the fully glycosylated monomers or dimers, respectively. (**A**) A representative immunoblot of DAT WT and its D301 mutants with or without CuP treatment. (**B**) Quantification of CuP-induced increases in dimer fraction and decreases in transport activity of DAT WT and its D301 mutants. Increased dimer fraction or decreased transport activity by CuP treatment was calculated from the control dimer fraction or transport activity without CuP treatment by subtracting its respective value after CuP treatment, respectively. Results are presented as mean ± SEM (n = 3). ** *p* < 0.01; *** *p* < 0.001 compared to DAT WT.

As shown in Figure 1, a unique aspartate residue in the EL3 of DAT (Asp301) or NET (Asp298) is proposed to stabilize the adjacent ion-pair (R304-E307 in DAT or R301-E304 in NET) interactions. To examine the significance of this aspartate in the regulation of dimerization and transport function, we substituted Asp301 in DAT with asparagine (the corresponding residue in SERT) or alanine to eliminate the proposed electrostatic interactions between Asp301 and Arg304 and performed an immunoblot analysis and transport assay for the mutants with and without CuP treatment, respectively. Compared to the WT, the substitutions dramatically reduced the influences of CuP treatment on the dimerization and transport activity of DAT (Figure 7A,B), suggesting that the elimination of the D301-R304 interactions significantly decreased the dimeric interface interactions, which, in turn, reduced their sensitivity to CuP oxidation. Noticeably, the cells stably expressing either D301A or D301N produced an extremely low level of DAT, compared to the WT. Although a small amount of fully glycosylated monomers and dimers of DAT in the total cell lysates of the D301A or D301N mutants was observed in the immunoblot analysis (Figure 7A), we were not successful in the detection of their immunoreactive bands on the cell surface in our biotinylation experiments. Nonetheless, the transport activity was determined to be 53.73 ± 2.57% of WT for D301A or 64.18 ± 4.12% of WT for D301N, respectively. Thus, we propose that the D301-R304 ionic interactions play an important role in stabilizing the conserved ion-pair R304-E307 interactions to form a salt bridge, which, in turn, enhances the interfacial interactions and subsequently increases the stability of DAT in the plasma membrane.

### 2.8. Effects of E322A Mutation on SERT Conformation

**Figure 8:**
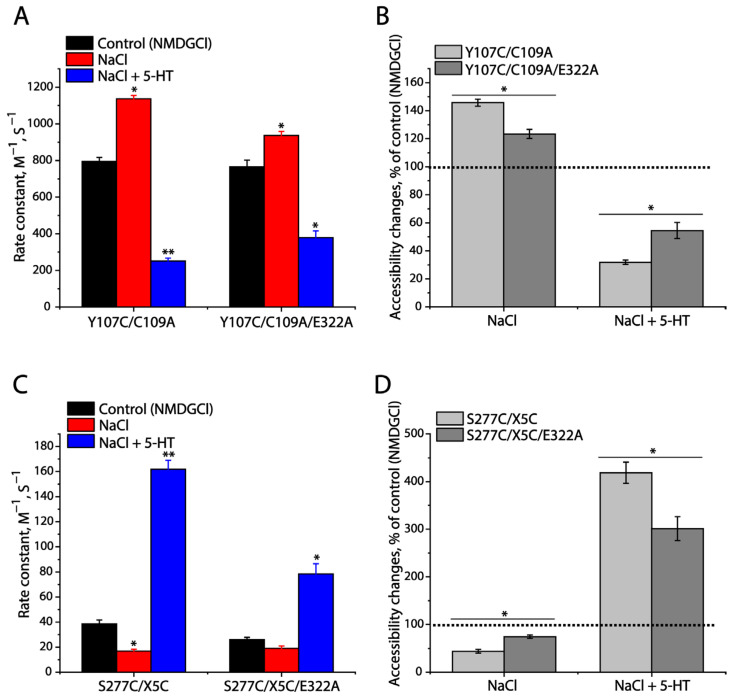
Effects of Asp322-to-Ala mutation on cysteine accessibility in both the extracellular and cytoplasmic pathways in SERT. (**A**) Rate constants of MTSET reactivity determined by the inhibition of APP^+^ uptake by the cells stably expressing Y107C/C109A or Y107C/C109A/E322A under various conditions (see Section 3 and Figure S1A,B). (**B**) Relative accessibility changes to the control (NMDGCl) in Y107C/C109A or Y107C/C109A/E322A. (**C**) Rate constants of MTSEA reactivity determined by the inhibition of ASP^+^ binding by the cells stably expressing S277C/X5C or S277C/X5C/E322A treated with 25 μg/mL digitonin (see Section 3 and Figure S1C,D). (**D**) Relative accessibility changes to the control (NMDGCl) in S277C/X5C or S277C/X5C/E322A. Results are presented as mean ± SEM (n = 3). * *p* < 0.05; ** *p* < 0.01 compared to the control (NMDGCl). The dotted lines in (**B**,**D**) show the control values, respectively.

As mentioned, the conserved ion-pair mutations of SERTs resulted in a significant increase in their cell surface expression but with an impaired specific transport activity. To determine whether the mutations influence the conformational changes in SERTs that are essential for substrate transport, we performed cysteine accessibility measurements, according to a well-established approach for measuring the accessibility of strategically positioned cysteine residues in both the extracellular and cytoplasmic permeation pathways to calculate the rate constants of reactivity with methanethiosulfonate (MTS) reagents [27]. The cysteine mutants selected for this study included Y107C/C109A in the extracellular pathway and S277C/X5C in the cytoplasmic pathway. As shown in Figure S1A, incubation with 2-(trimethylammonium)ethyl methanethiosulfonate bromide (MTSET) dramatically inhibited APP^+^ uptake by Y107C/C109A in a MTSET concentration-dependent manner. MTSET inhibition was sensitive to Na^+^ binding or substrate transport. Specifically, Na^+^ binding led to a shifting of the inhibition to a lower MTSET concentration range, compared to the control (NMDGCl, N-methyl-D-glucamine chloride), indicating that MTSET reactivity with Y107C/C109A became faster than the control (Figure S1A). In contrast, incubation with the substrate, 5-HT, in the presence of both Na^+^ and Cl^−^ ions, induced the inhibition, shifting to a higher MTSET concentration range, reflecting that the MTSET reactivity was slower than in the control (Figure S1A). Figure 8A shows the rate constants of MTSET reactivity, which were calculated from the IC_50_ values of the MTSET inhibition of APP^+^ uptake (Figure S1). In comparison with the rate constant of the control, Na^+^ binding increased MTEST reactivity, but 5-HT transport decreased it, indicating that Na^+^ stabilizes an outward-open conformation of SERT, while substrate transport stabilizes an outward-closed conformation, consistent with our previous observation [27]. On the other hand, mutation of the ion-pair residue Asp322 to Ala significantly impaired the effects of both Na^+^ binding and 5-HT transport on the MTSET inhibition of substrate transport (Figure S1B) and the rate constant MTSET reactivity (Figure 8A,B), indicating that the mutation slowed down the conformational changes in the extracellular pathway in SERT.

Similarly, for measuring the conformational changes in the cytoplasmic pathway in response to Na^+^ binding and substrate transport, we employed S277C/X5C to examine the effect of the Asp322-to-Ala mutation on the cytoplasmic cysteine Cys277 accessibility to 2-aminoethyl methanethiosulfonate hydrobromide (MTSEA) in digitonin-permeabilized cells. As shown in Figure S1C, compared to the control (NMDGCl), Na^+^ binding significantly decreased the rate constant of MTSEA reactivity, whereas substrate transport (5-HT in the presence of both Na^+^ and CI^−^) increased it (Figure 8C), indicating that Na^+^ binding stabilizes an inward-closed conformation in the cytoplasmic pathway while substrate transport stabilizes an inward-open conformation of SERT. These results, together with those in the extracellular pathway, are consistent with an alternating access mechanism in neurotransmitter transporters [27]. However, mutation of the ion-pair residue Asp322 to Ala resulted in a significant reduction in the effects of either Na^+^ binding or substrate transport, compared to that of the control (Figure 8C,D), indicating that the ion-pair mutation impaired the conformational changes in the cytoplasmic pathway.

### 2.9. Effects of the Ion-Pair Mutation on the Dimeric Interface Interactions of SERTs or DATs

**Figure 9:**
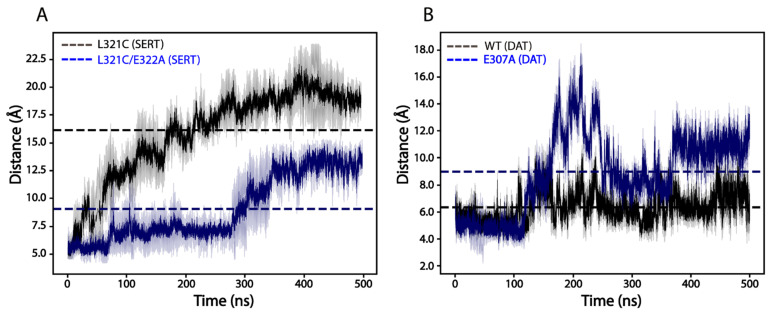
Time-course of the interactions between the cysteine pairs in SERT or DAT dimeric models during molecular dynamics simulations. (**A**) Distances between Cys321-Cys321′ in a dimeric model of SERT L321C (black) or SERT L321C/E322A (blue). (**B**) Distances between Cys306-Cys306′ in a dimeric model of DAT WT (black) or E307A (blue). Each simulation was performed three times (columns). The dotted lines represent average distances between the respective cysteine pairs in SERT or DAT dimeric models during 500 ns-long simulations.

To further compare the effects of the ion-pair mutations on the dimeric interface interactions between SERTs and DATs, we carried out 500 nanosecond (ns)-long molecular dynamics simulations of SERTs (L321C and L321C/E322A) and DATs (WT and E322A) based on our dimeric structural models with each protomer in an outward-open conformation. Figure 9 shows the time course of the distances between the respective cysteine pairs during simulations. The average distance between Cys321-Cys321′ in the dimeric model of the background construct L321C of SERT was estimated to be 16.15 ± 1.08 Å (Figure 9A). The ion-pair mutation E322A decreased the distance by approximately 2-fold to 9.08 ± 0.81 Å, indicating that disruption of the ion-pair interactions increased the dimeric interfacial interactions between SERT protomers. By comparison, the average distance between Cys306-Cys306′ in DAT WT or E307A was assessed to be 6.35 ± 0.49 Å or 8.98 ± 0.54 Å (a 1.4-fold increase compared to that in DAT WT), respectively (Figure 9B), showing that a similar mutation at the corresponding position resulted in a reduction in the dimeric interface interactions between DAT protomers. We should mention that the cysteine-pair distances measured from our simulations are larger than those we expected, especially those in SERT, probably due to the lack of high-resolution dimeric structures of either SERTs or DATs in various conformations and simulations of each transporter only in an outward-open conformation. Nevertheless, it is clear that disruption of the ion-pair interactions resulted in opposite effects on the dimeric interface interactions between SERTs and DATs, supporting the results obtained from our biochemical experiments.

## 3. Discussion

The monoamine transporters, SERT, DAT, and NET, have been demonstrated to form dimers and even higher-order oligomers by using various biochemical approaches [18,26,28]. CuP treatment enhances DAT dimerization through oxidative cross-linking of an endogenous cysteine residue, Cys306, in a proposed dimeric interface [20]. The results presented here show that replacement of the corresponding residue with a cysteine leads to oxidation-induced disulfide formation between SERT and NET protomers. It is unknown if dimerization or oligomerization of the monoamine transporters involves disulfide cross-linking in vivo. However, the fact that these disulfides readily form when cysteine is present at the equivalent position suggests that dimerization of the monoamine transporters may involve at least the face of the transporter proteins where the cysteine residue resides. In addition, earlier studies on the functional consequences of DAT or GlyT1 dimerization, combined with our observation of the relationship between CuP-induced dimerization and functional response in the present study, support the proposal that the monoamine transporters share a common mechanistic feature by which dimerization impairs their transport function.

**Figure 10:**
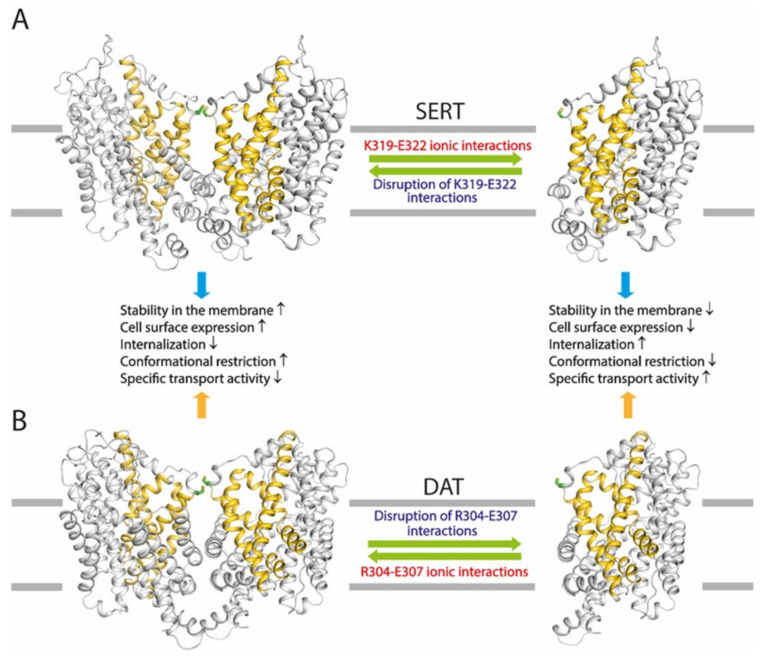
Schematic presentation of the effects of the conserved ion-pair on an equilibrium between SERT (**A**) and DAT (**B**) monomers and dimers on the cell surface and the proposed functional consequences. The conserved ion-pair is proposed to play a critical but divergent role in the regulation of equilibrium between monomers and dimers on the cell surface among the monoamine transporters. Disruption of the ion-pair interactions by mutations leads to opposite effects on an equilibrium of SERTs or DATs on the cell surface, consequently resulting in different functional responses. Structural models of the SERT or DAT monomer and dimer in outward-open conformations were generated as described in Section 4.3, respectively. The bundle domains (TMs 1, 2, 6, and 7) and other parts of the transporter proteins are colored golden and gray, respectively. The cysteine residues that are responsible for the oxidation-induced disulfide bond formation in a dimer are shown in sticks with a green color.

The present study demonstrates that a highly conserved intramolecular ion-pair at the EL3 connecting TM5 and TM6 plays a critical but different role in the regulation of the transport function of the monoamine transporters through their dimerization. The ion-pair residues have been proposed to form a salt bridge to stabilize the EL3 in a conformation that modulates the dimeric interface interactions [22]. Indeed, our biochemical experiments presented here indicate that disruption of the ion-pair interactions by mutations leads to a profound impact on the oxidation-induced cross-linking of all three monoamine transporters. Strikingly, the ion-pair mutations exert opposite effects among these transporters. Substitutions of the ion-pair residues with alanine cause a remarkably spontaneous cross-linking of SERT protomers, possibly due to an increase in the dimeric interface interactions in the SERT ion-pair mutants (Figure 10A). By contrast, similar substitutions result in a significant reduction in the oxidation-induced cross-linking of DAT or NET protomers, suggesting a decrease in the dimeric interface interactions in the DAT or NET mutants (Figure 10B). The opposite effects of the ion-pair mutations on the dimeric interface interactions among these transporters are supported by our molecular dynamics simulations that indicated that the ion-pair mutations remarkably reduced the average distance between two thiol groups of the cysteines Cys321-Cys321′ in a SERT dimer, but increased it of the cysteines Cys306-Cys306′ in a DAT dimer, compared to their respective control constructs.

On the cell surface, the transporters were proposed to be present in a dynamic equilibrium between monomers and dimers [29]. Alterations in the interfacial interactions between protomers may affect the equilibrium, which, in turn, alters transporter stability in the membrane and cell surface expression. Our results show that mutations of the ion-pair residues lead to a dramatic increase in SERT cell surface expression, suggesting that the increased interfacial interactions in these mutants shift the equilibrium towards more dimers that are expected to stabilize SERTs in the plasma membrane, and subsequently decrease their internalization (Figure 10A). On the other hand, similar mutations in DAT affect the equilibrium in an opposite direction, resulting in a shift towards more monomers that are expected to have a lower stability in the plasma membrane and a stronger internalization compared to that of dimers (Figure 10B).

The increased dimeric interface interactions caused by mutating the ion-pair residues result in an elevation of SERT cell surface expression, its specific transport activity, however, is significantly lower than its background construct (Figure 10A). It has been hypothesized that an increase in the interfacial interactions slows down the transport process because of the need to break the interfacial interactions for the rapid conformational conversions essential for substrate transport [23,24]. Our recent biochemical experiments indicated that the rapid conformational conversions caused by a large-scale repeated rotation of the bundle domain during substrate transport impaired the dimeric interface interactions, and subsequently reduced the oxidation-induced cross-linking of GlyT1 protomers [26], supporting the idea that a conformational restriction of substrate transport occurs in the dimeric quaternary structures of the NSS transporters. Similarly, the present study on cysteine accessibility provides direct evidence that an ion-pair mutation significantly decreases the effects of either Na^+^ or substrate transport on rate constants of the reactivity with MTS reagents in both the extracellular and cytoplasmic substrate permeation pathways, consistent with the proposal that an increase in the interface interactions results in a reduction in conformational transitions in SERT. In contrast, a decrease in the dimeric interface interactions by similar mutations in DAT reduces its cell surface expression and subsequently results in a remarkable increase in its specific transport activity (Figure 10B). Correspondingly, it can be interpreted by a higher flexibility of the bundle domain in the ion-pair mutants of DAT for conformational changes during the transport cycle than that of the WT.

The fact that different mutations in the conserved ion-pair residues in either SERT, DAT, or NET lead to effects, with different extents, on the dimerization and transport function provides direct evidence to support the existence of a dynamic equilibrium between monomers and dimers of the monoamine transporters on the cell surface, through which the transport function is precisely modulated by their dimerization. Apparently, the ion-pair in SERT likely tends to attenuate the dimeric interface interactions, and then reduce its dimerization so as to activate its transport function. By comparison, the corresponding ion-pair, possibly together with the additional ionic interactions between D301-R304 in DAT, works to reinforce the interfacial interactions between protomers, thus increasing their dimerization and impairing their transport activity. However, the conserved ion-pair interactions also regulate cell surface expression of the monoamine transporters through their dimerization which has been demonstrated to modulate transporter stability in the plasma membrane and internalization. The substrate transport ability of a given membrane transporter is believed to rely not only on its specific transport activity but also on its cell surface expression. Thus, it is reasonable to assume that optimization of the transport function of the monoamine transporters can be achieved through a potential compensatory mechanism that might be activated in response to the disrupted ion-pair interactions by regulating the equilibrium between monomers and dimers (Figure 10). Therefore, we propose functional regulation through their dimerization with similar mechanistic features but different molecular solutions among the monoamine transporters.

Despite many efforts of in vitro and in vivo studies, the physiological role of the dimerization or oligomerization of the monoamine transporters remains to be fully understood. Experiments with rat hippocampal slices demonstrated that SERT-mediated efflux of the preloaded substrate was significantly reduced by the phospholipase C agonist m-3M3FBS [30], which was later shown to reduce the amount of SERT oligomers in in vitro experiments [31]. In addition, a membrane lipid, phosphatidylinositol 4,5-bisphosphate (PIP2, the substrate of the phospholipase C), has been shown to regulate psychostimulant behaviors in *Drosophila melanogaster* through its interaction with DAT [32]. Interestingly, direct binding of PIP2 to SERT [30] and DAT [33] at different regions was shown by immunoprecipitation. PIP2 was also shown to interact with SERT at the lipid–membrane interface [30] and to stabilize the transporter dimers [34]. These fundamental works have revealed the physiological relevance of dimerization or oligomerization of the monoamine transporters and their potential implications for therapeutic development. The present study aims to increase the understanding of the mechanism of transporter dimerization and its significance in the regulation of transport function, which could assist in the development of novel therapeutic agents targeting transporter dimerization.

## 4. Materials and Methods

### 4.1. Materials

HEK 293T cells were from the American Type Culture Collection. Anti-FLAG M2 agarose gel, monoclonal anti-FLAG antibody, 3 x FLAG peptide, 4-[4-(dimethylamino) phenyl]-1-methylpyridinium (APP^+^), 4-[4-(dimethylamino) styryl]-N-methylpyridinium (ASP^+^), fluoxetine, GBR12909, and desipramine were purchased from Sigma-Aldrich (St. Louis, MO, USA). Sulfosuccinimidyl 2-(biotinamido) ethyl-1,3-dithiopropionate (sulfo-NHS-SS-biotin), streptavidin agarose gel, and Super Signal West Pico were from Thermo Fisher Scientific (Waltham, MA, USA). All other reagents were of analytical grade.

### 4.2. Mutagenesis and Stable Cell Line Preparation

The full-length cDNA encoding C-terminal FLAG-tagged human SERT (hSERT), human DAT (hDAT), or human NET (hNET) was amplified using respective expression plasmids in pcDNA3.1 as a template by PCR and inserted into the Xba I and BamH I sites of the lenti-EF-1α-BSD vector by Exnase II. The mutants used for the cross-linking study in this work were constructed in the lentiviral plasmids. All mutants were generated using the Mut Express II Fast Mutagenesis Kit (Vazyme, Nanjing, China) and confirmed by full-length DNA sequencing.

The lentivirus and HEK 293T stable cell lines for hSERT, hDAT, or hNET and their mutants were prepared as described previously [35]. The stable cell lines were maintained in DMEM supplemented with 10% fetal bovine serum, 100 units/mL penicillin, 100 μg/mL streptomycin, and 12 μg/mL blasticidin S at 37 °C in a humidified 5% CO_2_ incubator, and then plated in 12-well culture plates. The stable cell lines expressing hSERT, hDAT, or hNET were confirmed by immunoblot analysis for the respective transporter expression. Protein concentration was determined with the Micro BCA protein assay reagent kit (Thermo Fisher, Waltham, MA, USA).

### 4.3. Homology Modeling and Dimer Docking of SERT, DAT, and NET

Homology models of hDAT and hNET were generated with PyMod version 3.0.2 (https://schubert.bio.uniroma1.it/pymod/index.html, accessed on 5 October 2023) [36] based on an outward-facing structure (2.89 Å resolution, PDB ID, 4XP1) of the *Drosophila melanogaster* DAT [13], as described previously [37]. The cryo-electron microscopy structure of hSERT (3.30 Å resolution, PDB ID, 7LIA) [11] and models of hDAT or hNET in outward-open conformations were used for generating transporter dimers using the multimer docking module in ClusPro 2.0 (https://cluspro.bu.edu/, accessed on 5 October 2023) [38,39,40,41], respectively. Dimer docking was performed by using Cys321 of hSERT, Cys306 of hDAT, or Cys303 of hNET as an anchoring point, which generated more than 30 dimer models for each transporter. One pose with the best score was selected and exported into PyMOL version 2.5.5 (Schrödinger Inc., New York, NY, USA) for visualization.

### 4.4. Oxidative Cross-Linking

HEK 293T cells stably expressing hSERT, hDAT, hNET WT, or their mutants in a 6-well plate were washed twice with HEPES buffered saline (10 mM HEPES, pH 7.4, 1.2 mM MgSO_4_, 2 mM K_2_SO_4_, 10 mM glucose, and 150 mM NaCl) and incubated with copper phenanthroline (CuP, a mixture of CuSO_4_ and 1,10-phenanthroline) at the indicated concentrations at 22 °C for 15 min, as described previously [42,43]. The cross-linking reaction was stopped by 2 × rapid washing with HEPES buffered saline and incubating with 10 mM N-ethylmaleimide for 15 min, followed by three more washes. The cells were lysed and the lysates in a non-reducing sample buffer were then assayed for dimerization by immunoblot analysis. To examine DTT cleavage of CuP-induced disulfate bond formation, cell lysates were incubated with 100 mM DTT at 22 °C for 1 h prior to immunoblot analysis.

### 4.5. Transport Assay

APP^+^ or ASP^+^ transport by hSERT or hDAT and hNET was measured in polylysine-coated 12- or 96-well plates, as described previously [44]. In brief, the cells were washed twice with 500 μL KRH buffer containing 20 mM HEPES, pH 7.4, 120 mM NaCl, 1.3 mM KCl, 2.2 mM CaCl_2_, 1.2 mM MgSO_4_, and 0.1% (*w*/*v*) glucose. APP^+^ or ASP^+^ uptake was assayed by adding 500 μL KRH buffer containing 2 μM APP^+^ for hSERT or 6 μM ASP^+^ for hDAT or hNET and incubating at 22 °C for 5 min. Excess APP^+^ or ASP^+^ was then removed by rapid washing three times with 500 μL KRH buffer. The extent of APP^+^ or ASP^+^ accumulated in the cells was determined by confocal imaging analysis with the LSM 900 confocal microscope (Zeiss, Oberkochen, Germany) and fluorescence spectrometry with the Infinite 200 Pro Microplate Reader (Tecan, Grödig, Austria). Nonspecific APP^+^ or ASP^+^ transport by the cells was determined in the presence of 100 μM fluoxetine, GBR12909, or desipramine, and subtracted to give APP^+^ or ASP^+^ uptake by hSERT, hDAT, or hNET, respectively.

### 4.6. Immunoblot Analysis

The cells stably expressing C-terminal Flag-tagged hSERT, hDAT, or hNET in a 6-well plate were lysed with 250 μL RIPA buffer containing 50 mM Tris, pH 7.4, 5 mM EDTA, 150 mM NaCl, 1% Triton X-100, and the detergent extracts (60 μg of total proteins per well) were separated by a 10% SDS-polyacrylamide gel, transferred to a PVDF membrane (Bio-Rad, Hercules, CA, USA), and probed with anti-FLAG monoclonal M2 antibody (1:1000). A horseradish peroxidase-conjugated anti-mouse IgG (1:10,000) was used to visualize the signal by Super Signal West Pico with a UVP Biochemi II imaging system (Analytik Jena, Upland, CA, USA).

### 4.7. Biotinylation

Cell surface expression of hSERT, hDAT, hNET, or their mutants was determined using membrane-impermeant biotinylation reagent sulfo-NHS-SS-biotin, as described previously [45]. HEK 293T cells stably expressing WT or mutants of hSERT, hDAT, or hNET (100% confluence in a medium-size culture dish containing 1 × 10^8^ cells) were treated twice with sulfo-NHS-SS-biotin on ice for 20 min. After labeling, the cells were rinsed with 100 mM glycine in phosphate-buffered saline (PBS) containing 137 mM NaCl, 2.7 mM K_2_SO_4_, 4.3 mM Na_2_HPO_4_, and 1.4 mM KH_2_PO_4_, pH 7.4 on ice for 20 min to quench excess sulfo-NHS-SS-biotin. The cells were then lysed, and the biotinylated proteins were recovered using streptavidin–agarose beads in an overnight incubation at 4 °C with gentle agitation. The beads were washed, and the biotinylated proteins were eluted with 100 μL SDS-PAGE sample buffer. Samples were applied to a 10% SDS-polyacrylamide gel and visualized by immunoblot analysis. The transporters were detected using anti-FLAG monoclonal M2 antibody (1:1000) against the FLAG epitope tag at the C-terminus of hSERT, hDAT, or hNET. Immunoreactive bands were visualized by chemiluminescence, and cell surface expression was quantified using a UVP Biochemi II imaging system.

### 4.8. Reversible Biotinylation

An internalization (endocytosis) assay for hSERT or hDAT was performed by reversible biotinylation, as described previously [46,47]. In brief, HEK 293T cells stably expressing hSERT, hDAT, or their mutants were cooled rapidly to 4 °C to inhibit endocytosis, by washing with ice-cold PBS, and the cell surface proteins were biotinylated with sulfo-NHS-SS-biotin at 4 °C for 20 min. After the excessive biotinylating reagent was quenched with 100 mM glycine, hSERT or hDAT, endocytosis was initiated by incubating the cells with a prewarmed buffer at 37 °C for 30 min. At the end of incubation, the endocytosis was stopped by adding an ice-cold PBS buffer. The cells were then incubated twice with 50 mM sodium 2-mercaptoethanesulfonate (MesNa), a reducing agent in an ice-cold cleaving buffer (50 mM Tris-HCl, pH 8.9, 50 mM MesNa, and 100 mM NaCl) for 20 min to cleave the disulfide bond formed between the sulfo-NHS-SS-biotin and cell surface proteins. To define total biotinylated transporters, one biotinylated sample was not subjected to MesNa reduction and directly processed for solubilization with RIPA buffer followed by isolation by streptavidin beads. To define MesNa reduction efficiency, another sample was treated with MesNa immediately after biotinylation at 4 °C. After the samples were solubilized with RIPA buffer, the biotinylated transporter proteins were isolated using streptavidin beads and subjected to immunoblot analysis.

### 4.9. Immunofluorescence Staining

The immunofluorescence staining was performed with HeLa cells stably expressing SERT (L321C or E322A/L321C) or DAT (WT or E307A). Briefly, 1 day before fixation, the cells were seeded onto poly-D-lysine-coated coverslips in 12-well plates. Cells were fixed with 4% paraformaldehyde for 15 min, followed by permeabilization with 0.5% Triton X-100 for 20 min, and then application of primary antibodies (rabbit anti-Flag, 1/2000 and mouse anti-calnexin, 1/400) overnight at 4 °C. Treatment with secondary antibodies conjugated to fluorophores Alexa 488 or 594 for 1 h was followed by mounting of coverslips to charged slides. Immunofluorescence images were captured using a Zeiss LSM 900 confocal microscope (Zeiss, Oberkochen, Germany).

### 4.10. Cell-Based SERT Cysteine Accessibility Measurements

The cysteine accessibility measurement protocol was adapted from previous work [44]. Conformational changes in SERT were measured using the accessibility of cysteine residues placed in the extracellular (Y107C) and cytoplasmic (S277C) permeation pathways, respectively. For measurement of extracellular pathway accessibility, measurements were performed with intact cells stably expressing SERT Y107C/C109A growing on poly-D-lysine-coated coverslips in 24-well plates. For cytoplasmic pathway accessibility, measurements were made with digitonin-permeabilized cells stably expressing SERT S277C/X5C. Transport or binding assay was performed using APP^+^ or ASP^+^, respectively. In both cases, accessibility was measured by the rate of cysteine reactivity with MTSET or MTSEA, as described previously [48]. NMDG was used to replace Na^+^. The MTSET or MTSEA concentration causing half-maximal inactivation was determined and used to calculate the rate constant for cysteine modification, as described previously [49,50].

### 4.11. Molecular Dynamics Simulations

Molecular dynamics simulations of SERT and DAT dimeric models were performed using Amber 22 (San Francisco, CA, USA) [51,52]. Each protomer included one Cl^−^ and two Na^+^ ions at the Na1 and Na2 sites. Protein dimers were embedded in a 1-palmitoyl-2-oleoyl-glycero-3-phosphocholine (POPC) lipid bilayer. The ff19SB force field [53] was used for calculating system force field parameters. Solvation was performed with the TIP3P water model [54]. Sodium and chloride ions were added to achieve 150 mM NaCl concentration. Temperature was kept constant at 300 K and pressure was set to 1 atmosphere. Molecular simulations were conducted for a 500 ns period. Each simulation comprised 250 million steps (0.002 femtosecond/step). Analysis of the simulations was carried out using Amber Tools 23 [55,56].

### 4.12. Data Analysis

Nonlinear regression fits of experimental and calculated data were performed with Origin 2021 version 9.8.0.200 (Origin Lab, Northampton, MA, USA). The statistical analysis given was from multiple experiments. Data with error bars in the figures represent the mean ± SEM for at least three experiments as indicated, respectively. Statistical analysis was performed using Student’s paired *t*-tests.

## 5. Conclusions

The present study characterized a highly conserved intramolecular ion-pair in the regulation of transport function through dimerization of the monoamine transporters, by using biochemical and biophysical approaches. Our results indicated that disruption of the ion-pair interactions by mutations led to critical but divergent effects on dimerization and transport function among these transporters. We proposed that the transport activities of the monoamine transporters are modulated by the equilibrium between monomers and dimers that is expected to regulate both their specific transport activities and cell surface expression levels. The present study provided new insights into structural elements regulating the transport function of the monoamine transporters through their dimerization.

## Data Availability

The data presented in this study are available on request from the corresponding author.

## Supplementary Materials

The following supporting information can be downloaded at: https://www.mdpi.com/article/10.3390/ijms25074032/s1.
