# Supplementary material for: A Conserved Intramolecular Ion-Pair Plays a Critical but Divergent Role in Regulation of Dimerization and Transport Function among the Monoamine Transporters
Source: Int J Mol Sci. 2024 Apr 4;25(7):4032. doi: 10.3390/ijms25074032 (PMC11011927; doi:10.3390/ijms25074032)
Supplement: Supplementary file 1 [file ijms-25-04032-s001.zip › ijms-2874563-supplementary.pdf]

Table S1. Kinetic parameters of APP^+ uptake by SERT WT and its mutants with or without CuP treatment.

	**APP^+**	
	**K_m (μM)**	**V_{max} (AFU)**
**WT**	2.40 ± 0.05	24.32 ± 1.86
**L321C**	2.43 ± 0.08	22.31 ± 1.27
**L321C + CuP**	2.32 ± 0.17	8.47 ± 0.27*
**K319A/L321C**	2.45 ± 0.08	25.59 ± 1.66
**K319A/L321C + CuP**	2.54 ± 0.20	17.65 ± 1.54*
**E322A/L321C**	2.59 ± 0.10	26.83 ± 2.33
**E322A/L321C + CuP**	2.62 ± 0.08	19.97 ± 2.25*

APP^+ uptake was measured over a range of APP^+ concentrations (0.01 – 10 μM) with cells stably expressing SERT WT or its mutants as described under "Materials and Methods". For CuP treatment, CuP at a final concentration of 100 μM was added to the cells for 10 min at 22 °C and then washed away. Results are shown as mean ± SEM averaged from three independent experiments. * $p < 0.05$ compared to the respective mutant without CuP treatment.

Table S2. Kinetic parameters of ASP^+ uptake by DAT WT and its mutants with or without CuP treatment.

	ASP^+	
	K_m (μM)	V_{max} (AFU)
**WT**	4.28 ± 0.18	42.12 ± 3.13
**WT + CuP**	3.96 ± 0.29	23.91 ± 0.89*
**R304A**	4.39 ± 0.21	33.76 ± 1.43
**R304A + CuP**	4.18 ± 0.28	23.54 ± 1.75*
**E307A**	4.09 ± 0.19	31.18 ± 4.52
**E307A + CuP**	3.92 ± 0.42	26.41 ± 1.83*

ASP^+ uptake was measured over a range of ASP^+ concentrations (0.01 – 10 μM) with cells stably expressing DAT WT or its mutants as described under "Materials and Methods". For CuP treatment, CuP at a final concentration of 100 μM was added to the cells for 10 min at 22 °C and then washed away. Results are shown as mean ± SEM averaged from three independent experiments. * $p < 0.05$ compared to the respective mutant without CuP treatment.

Table S3. Kinetic parameters of ASP^+ uptake by NET WT and its mutants with or without CuP treatment.

	ASP^+	
	K_m (μM)	V_{max} (AFU)
**WT**	3.10 ± 0.08	63.82 ± 3.08
**K303C**	3.35 ± 0.11	60.60 ± 2.20
**K303C + CuP**	3.20 ± 0.19	31.67 ± 1.66*
**R301A/K303C**	3.33 ± 0.16	56.97 ± 3.10
**R301A/K303C + CuP**	3.55 ± 0.19	38.74 ± 1.77*
**E304A/K303C**	3.36 ± 0.18	47.15 ± 2.45
**E304A/K303C + CuP**	3.22 ± 0.17	40.07 ± 1.45*

ASP^+ uptake was measured over a range of ASP^+ concentrations (0.01 – 10 μM) with cells stably expressing NET WT or its mutants as described under "Materials and Methods". For CuP treatment, CuP at a final concentration of 100 μM was added to the cells for 10 min at 22 °C and then washed away. Results are shown as mean ± SEM averaged from three independent experiments. * $p < 0.05$ compared to the respective mutant without CuP treatment.

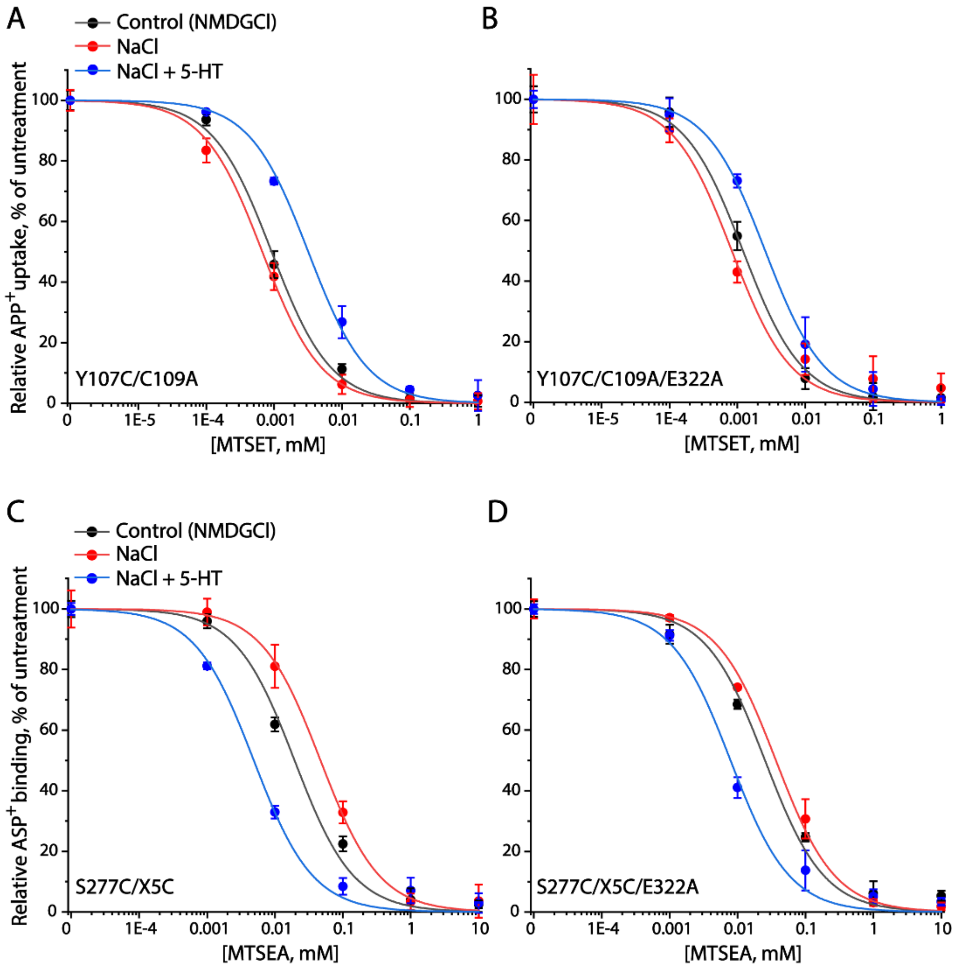

Figure S1. MTSET or MTSEA accessibility measurements in SERT. (A, B) MTSET accessibility in the extracellular substrate permeation pathway. MTSET inhibition of APP$^+$ uptake was measured in the intact cells stably expressing Y107C/C109A or Y107C/C109A/E322A by incubating with MTSET at a range of concentrations (0 -1 mM) in HEPES buffer containing 25 mM HEPES, pH 7.4 and 150 mM NMDGCl, 150 mM NaCl, or 150 mM NaCl plus 10 µM 5-HT for 15 min at 22 °C. After washing to remove excess MTSET, APP$^+$ uptake was measured as described under Section 3. (C, D) MTSEA accessibility in the cytoplasmic substrate pathway. MTSEA inhibition of ASP$^+$ binding was measured in the digitonin-permeabilized cells expressing S277C/X5C or S277C/X5C/E322A. The cells were incubated with 25 µg/mL digitonin for 5 min in HEPES buffer containing 25 mM HEPES, pH 7.4 and 150 mM NMDGCl, 150 mM NaCl, or 150 mM NaCl plus 10 µM 5-HT and then with MTSEA at a range of concentrations (0 – 10 mM) in the presence of digitonin for another 15 min at 22 °C. After washing to remove excess MTSEA and digitonin, ASP$^+$ binding was measured as described under Section 3. All graphs show representative experiments. These experiments were repeated twice with similar results.
